# Global, regional, and national burden of IHD attributable to PM pollution aged 70 and above: an age-period-cohort modeling and frontiers analysis study

**DOI:** 10.3389/fpubh.2025.1573599

**Published:** 2025-06-04

**Authors:** Ke-Jie He, Haitao Wang, Xu Liu, Rongying Yang, Guoyu Gong

**Affiliations:** ^1^The Quzhou Affiliated Hospital of Wenzhou Medical University, Quzhou People's Hospital, Quzhou, Zhejiang, China; ^2^The School of Clinical Medical Sciences, Southwest Medical University, Luzhou, Sichuan, China; ^3^Department of Neurology, The Second Affiliated Hospital of Xuzhou Medical University, Xuzhou, China; ^4^Key Laboratory of Medical Electrophysiology, Ministry of Education and Medical Electrophysiological Key Laboratory of Sichuan Province (Collaborative Innovation Center for Prevention of Cardiovascular Diseases), Institute of Cardiovascular Research, Southwest Medical University, Luzhou, Sichuan, China; ^5^School of Medicine, Xiamen University, Xiamen, China

**Keywords:** ischemic heart disease, global burden of disease, particulate matter pollution, disability adjusted life years (DALYs), age-period-cohort, socio-demographic index

## Abstract

**Background:**

Particulate matter (PM) pollution is a significant risk factor for ischemic heart disease (IHD). This study evaluates the global, regional, and national burden of IHD attributable to PM pollution from 1990 to 2021, quantifies key contributing factors, and projects trends to 2044, with a focus on regional disparities and population aging.

**Methods:**

Using data from the Global Burden of Disease (GBD) 2021 study, we analyzed trends in IHD-related disability-adjusted life years (DALYs) and mortality attributable to PM pollution. Joinpoint regression assessed long-term trends, Age-Period-Cohort modeling evaluated demographic drivers, and decomposition analysis identified the contributions of population growth, aging, and epidemiological changes. Frontier analysis compared observed DALY rates with the lowest achievable rates based on socio-demographic index (SDI). Future trends were projected using the Nordpred model.

**Results:**

From 1990 to 2021, global age-standardized DALY rates for IHD attributable to PM pollution decreased by −1.51% annually, but absolute DALYs increased due to population aging and growth. High SDI regions saw significant declines in DALY rates (−4.75% annually), while Low SDI regions experienced negligible change (0.01%). Population growth contributed to a 183.57% increase in global DALYs, but epidemiological improvements reduced the burden by 89.29%. Frontier analysis revealed substantial unrealized potential for reducing the IHD burden, particularly in Middle SDI regions. Projections to 2044 indicate that while DALY rates will decline, total DALYs will increase among individuals aged over 70, especially in Low and Low-middle SDI regions.

**Conclusions:**

This study highlights substantial progress in reducing the IHD burden attributable to PM pollution, particularly in High SDI regions. However, disparities remain, especially in Low and Low-middle SDI regions, where the aging population and insufficient healthcare infrastructure exacerbate the burden. The rising IHD burden among the older adult underscores the need for targeted interventions, including stricter air quality regulations, enhanced healthcare access, and policies that specifically address vulnerable populations. Strengthening healthcare systems and air pollution controls in these regions is critical to mitigating the growing IHD burden in the coming decades.

## 1 Introduction

Ischemic heart disease (IHD) is one of the leading causes of death and disability worldwide (1, 2), with its burden increasingly linked to environmental risk factors, including particulate matter (PM) pollution (3). PM pollution, particularly fine particulate matter (PM_2.5_), has been widely recognized as a significant contributor to cardiovascular diseases (4), with numerous studies demonstrating its association with increased morbidity and mortality due to IHD (5–7). The adverse health effects of PM pollution arise from its ability to penetrate the respiratory and circulatory systems (8), triggering systemic inflammation (9), oxidative stress (10), and endothelial dysfunction (11), which collectively accelerate the development of atherosclerosis (12, 13) (a major precursor of IHD). PM_2.5_ refers to particulate matter that is 2.5 μm or smaller in diameter, which can deeply penetrate the lungs and enter the bloodstream, whereas PM_10_ includes particles with a diameter of 10 μm or smaller (14). Both of these particle sizes have been linked to cardiovascular diseases, though their health effects may vary due to differences in their ability to reach the circulatory system. However, PM_2.5_ rarely exists in isolation and often interacts with other pollutants such as PM_10_, nitrogen dioxide (NO_2_), and ozone (O3), further exacerbating the risk of IHD (15). Studies have shown that combined exposure to these pollutants leads to synergistic effects, enhancing the inflammatory response, oxidative stress, and vascular dysfunction. For example, long-term exposure to both PM_2.5_ and O3 in Tehran has been linked to an increased burden of IHD, with PM_2.5_ alone contributing significantly to mortality from IHD, as estimated to account for 19.8–24.1% of IHD-related deaths. Additionally, the combined exposure to PM_2.5_ and O3 was associated with increased mortality rates from cerebrovascular diseases and respiratory conditions (16). This complex interplay of pollutants contributes to a heightened cardiovascular burden, making it crucial to consider the combined exposure to multiple air pollutants in understanding the full extent of the IHD risk attributable to air pollution.

Despite extensive research, critical knowledge gaps persist regarding the global, regional, and national burden of IHD attributable to PM pollution. While short-term exposure to PM pollution has been shown to exacerbate cardiovascular events, the long-term effects of sustained exposure, particularly to fine particulate matter (PM_2.5_), have not been fully understood. Long-term PM_2.5_ exposure has been linked not only to respiratory diseases and cardiovascular issues but also to neurological disorders (17).

The rationale for this study stems from the urgent need to address the unequal distribution of the IHD burden across different socio-demographic index (SDI) regions. Factors such as continued global population growth, aging, and urbanization are likely to exacerbate PM pollution exposure in many parts of the world (18, 19). Furthermore, while high-income regions have made significant progress in reducing air pollution levels and improving cardiovascular health (20), low-income and middle-income countries continue to face severe pollution challenges (20, 21), compounded by weaker healthcare systems and less stringent environmental regulations. Thus, a comprehensive analysis of the global, regional, and national burden of IHD attributable to PM pollution, as well as projections of future trends, is vital for informing public health strategies and policy interventions aimed at reducing this preventable disease burden.

This study aims to provide a comprehensive analysis of the burden of IHD attributable to PM pollution by quantifying global, regional, and national trends in IHD-related disability-adjusted life years (DALYs) and mortality from 1990 to 2021, identifying the contributions of population growth, aging, and epidemiological changes to the observed burden, evaluating the unrealized potential for reducing IHD burden by comparing observed DALY rates with the lowest achievable rates based on SDI using frontier analysis, and forecasting future trends in IHD burden attributable to PM pollution through 2044 to inform targeted public health interventions. By addressing these objectives, this study seeks to fill critical evidence gaps, provide actionable insights for policymakers, and support the development of equitable strategies to mitigate the burden of IHD globally.

## 2 Materials and methods

### 2.1 Overview

This study utilized comprehensive data from the Global Burden of Disease (GBD) 2021 study, managed by the Institute for Health Metrics and Evaluation (IHME). The GBD study provides annual updates on global health metrics, including a wide range of diseases, mortality, DALYs, and associated risk factors, covering global, regional, and country-specific trends (22, 23). This study focuses on the burden of IHD attributable to PM pollution, analyzing data from 1990 to 2021 and projecting future trends through 2044.

### 2.2 Data sources

We extracted data on IHD-related mortality and DALYs from the Global Burden of Disease Collaborative Network and GBD Study 2021 results, publicly available through the IHME's online data visualization tool (https://vizhub.healthdata.org/gbd-results/). These datasets provide detailed information on age-standardized mortality and DALYs related to IHD, categorized by age group, sex, year, and SDI region. Our analysis specifically focused on IHD attributable to PM pollution from 1990 to 2021, with future projections to 2044. Additionally, country-level data were used to explore regional disparities and trends, enabling us to assess differences in IHD burden across various SDI categories and geographic regions, highlighting areas with the highest and lowest burden.

### 2.3 Analytical methods

To investigate temporal trends, regional variations, and projections for the future burden of IHD attributable to PM pollution, we employed the following statistical and modeling techniques:

(1) **Joinpoint regression analysis:** This method was used to identify significant changes in the trends of IHD-related DALYs and mortality attributable to PM pollution over time. Joinpoint regression identifies specific points, known as joinpoints, where a significant shift in the annual percent change (APC) occurs, indicating periods of acceleration or deceleration in the burden. The method also calculates the average annual percent change (AAPC) across the entire study period to provide a comprehensive understanding of long-term trends. We applied this analysis both at the global level and across different SDI regions to capture regional disparities in burden growth.
(2) **Age-Period-Cohort (APC) modeling:** This method was utilized to disentangle the distinct contributions of aging, specific time periods, and birth cohorts to trends in IHD incidence and mortality attributable to PM pollution. APC models allow for the separation of three key effects: age effects, which represent the influence of aging on disease risk; period effects, which reflect changes in disease risk over time due to factors such as environmental conditions, healthcare improvements, and societal changes; and cohort effects, which capture variations in risk among individuals born in different time periods. By applying this method globally and across various SDI regions, we were able to evaluate how demographic shifts, healthcare advancements, and environmental factors contributed to changes in IHD burden. This approach provided insight into the temporal and cohort-specific drivers of disease risk.
(3) **Decomposition analysis:** This method was employed to quantify the relative contributions of population growth, aging, and epidemiological changes to the increase in IHD-related DALYs attributable to PM pollution. Epidemiological changes encompass factors such as urbanization and changes in lifestyle. The analysis was conducted globally and further stratified by SDI regions, enabling an examination of how these factors differed across various socio-economic contexts. By disaggregating the contributions, this approach provided insights into the primary drivers behind the increasing IHD burden linked to PM pollution.
(4) **Frontiers analysis:** This method was used to assess the unrealized potential for reducing IHD-related DALYs attributable to PM pollution based on each country's SDI. The frontier represents the lowest achievable DALYs rate for a given SDI, serving as a benchmark for comparison. Countries were plotted based on their actual DALY rates and SDI levels, and the gap between the frontier and a country's observed DALYs rate indicated the potential for improvement. We categorized countries with the largest gaps from the frontier to highlight those with the most significant room for reducing IHD burden. This analysis was applied globally and across SDI regions to evaluate how much a country's IHD burden could be reduced if it achieved frontier performance. Additionally, we identified countries in Low SDI regions that were closest to the frontier, indicating that they were already maximizing their potential given their current socio-economic status, as well as countries in High SDI regions that still had significant room for improvement despite higher development levels.
(5) **Nordpred prediction model:** To forecast the future burden of IHD attributable to PM pollution through the year 2044, we employed Nordpred prediction model. These models utilize historical data to predict future trends, incorporating both current and projected changes in demographic factors such as population aging and growth. In addition, shifts in epidemiological risk factors, such as lifestyle changes and improvements in healthcare, were also accounted for. The projections provide estimates of the future number of DALYs and DALY rates, broken down by age group, sex, and SDI levels. A particular focus was placed on the older adult population (those aged 70 and above), as this group is expected to experience the sharpest increase in IHD burden over time.

### 2.4 Socio-demographic index (SDI)

The SDI is a composite metric that reflects a region's development level, incorporating income per capita, educational attainment, and fertility rates (24, 25). It is calculated using these three key indicators, allowing countries and regions to be classified into five SDI categories: Low, Low-middle, Middle, High-middle, and High. This classification facilitates comparisons of disease burden and health trends across regions with varying levels of socio-economic development, offering a more precise understanding of global health disparities.

### 2.5 Statistical analyses

To account for the variability in DALY rates and model predictions, we assumed that these rates followed log-normal distributions. To estimate the uncertainty of the predicted increases in DALYs and mortality rates, we employed bootstrap resampling methods, generating 1,000 draws to calculate 95% confidence intervals (CIs). All statistical analyses were performed using Python version 3.7.3, with a significance threshold set at *p* < 0.05. Details of all of the above methods can be found in Supplementary material 1.

## 3 Results

### 3.1 Deaths and DALYs of IHD attributable to PM pollution in 2021

**Figure 1:**
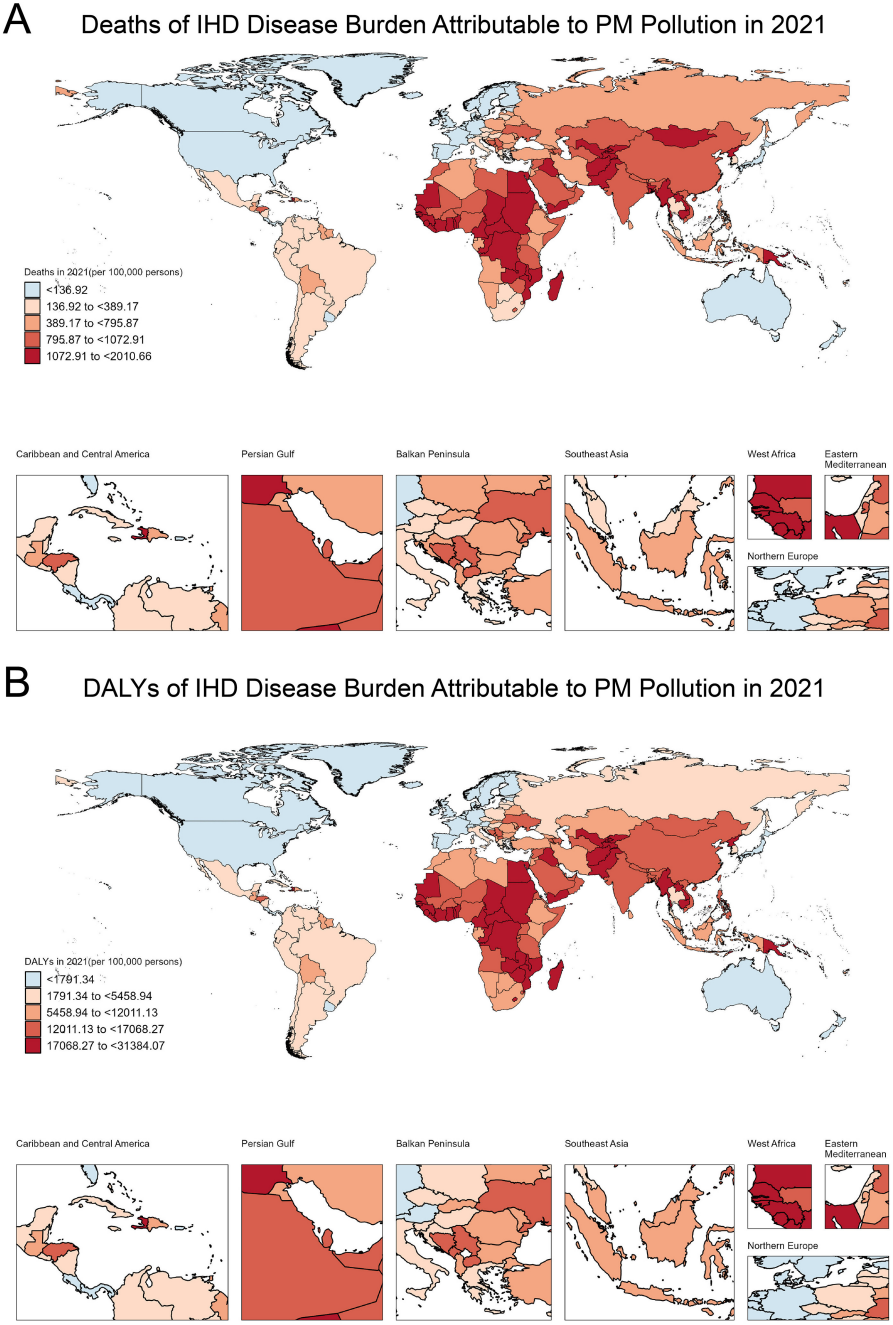
Global distribution of deaths and DALYs due to IHD attributable to PM pollution in 2021. **(A)** Deaths of IHD disease burden attributable to PM pollution in 2021 (per 100,000 persons). **(B)** DALYs of IHD disease burden attributable to PM pollution in 2021 (per 100,000 persons).

In 2021, the global distribution of deaths related to IHD attributable to PM pollution exhibited significant regional disparities (Figure 1A). Based on death rate, countries were categorized into five groups, ranging from fewer than 136.92 deaths per 100,000 to as high as 2,010.66 deaths per 100,000. Countries with the lowest death rate (< 136.92/100,000) included Spain, France, Norway, and Switzerland. Countries with slightly higher but still relatively low death rate (136.92/100,000–389.17/100,000) included Chile, Peru and Mexico. The middle-range death rate category (389.17/100,000–795.87/100,000) encompassed countries such as Poland, Guyana, and Namibia. Higher death rate (795.87/100,000–1,072.91/100,000) was observed in countries like Morocco, China, and Mali, while the highest death rate (1,072.91/100,000–2,010.66/100,000) was found in countries such as Samoa, Cambodia and Gambia.

Similarly, the 2021 distribution of DALYs, which reflect both mortality and morbidity, showed marked regional differences (Figure 1B). Countries with the lowest DALYs (< 1,791.34/100,000) included Iceland, France, and Canada. Countries with slightly higher DALYs (1,791.34/100,000–5,458.94/100,000) included Thailand, Poland, and Mexico, while the middle-range category (5,458.94/100,000–12,011.13/100,000) included Fiji, Morocco, and Tonga. Countries with moderately high DALYs (12,011.13/100,000–17,068.27/100,000) included Oman, Nepal, and Burundi. The highest DALYs (17,068.27/100,000–31,384.07/100,000) were observed in Sudan, Cameroon, and Samoa. The specific information of deaths in Supplementary material 2. The specific information of DALYs in Supplementary material 3.

### 3.2 Trends in DALYs of IHD attributable to PM pollution from 1990 to 2021 across SDI levels

**Figure 2:**
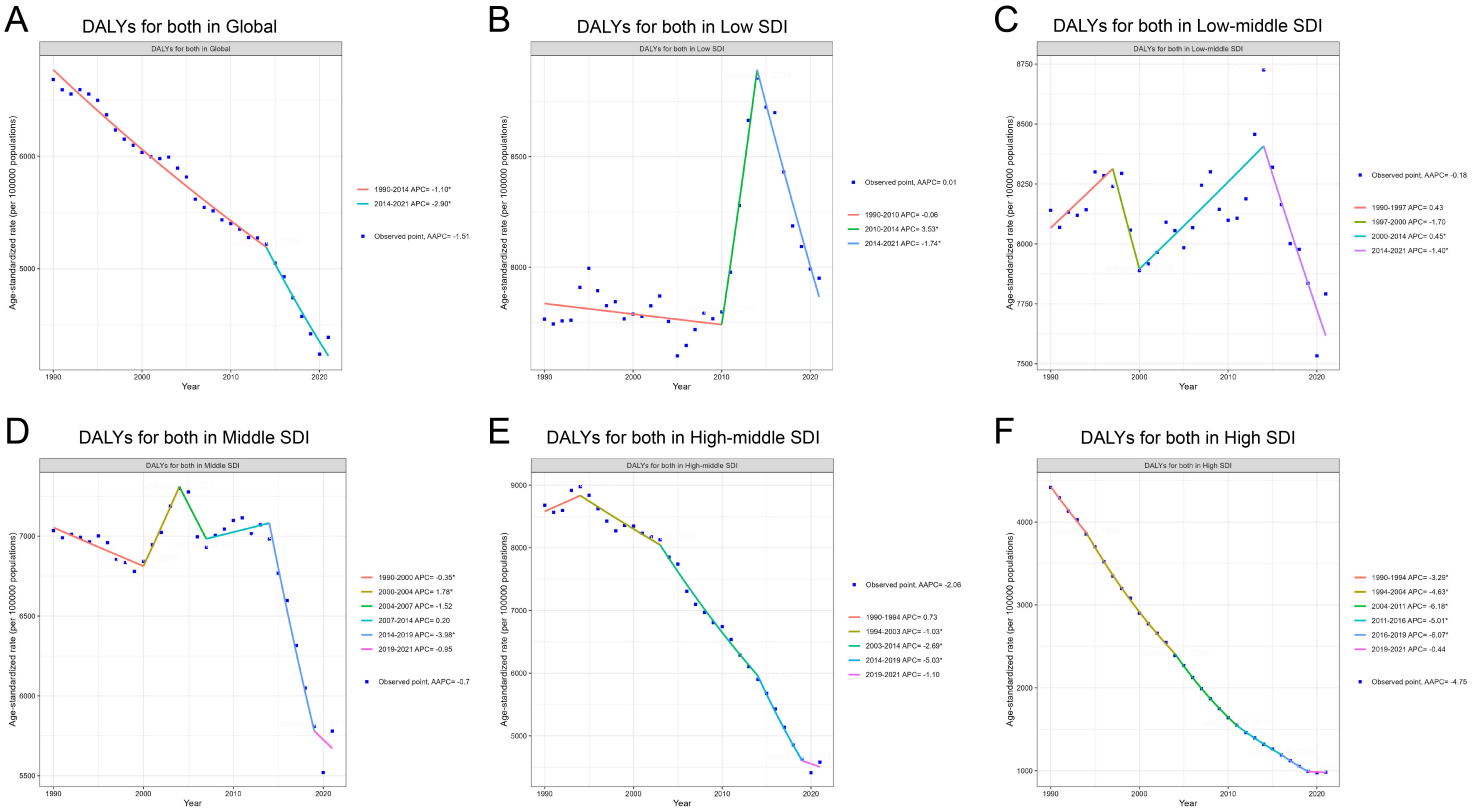
Trends in DALYs for IHD attributable to PM pollution from 1990 to 2021 across different SDI levels. **(A)** DALYs for both in Global. **(B)** DALYs for both in Low SDI. **(C)** DALYs for both in Low-middle SDI. **(D)** DALYs for both in Middle SDI. **(E)** DALYs for both in High-middle SDI. **(F)** DALYs for both in High SDI.

At the global level, there has been a consistent decline in the age-standardized DALY rates due to IHD attributable to PM pollution, with an overall average annual percentage change (AAPC) of −1.51%. Two distinct periods of change can be observed: from 1990 to 2014, the annual percentage change (APC) was −1.10%, followed by a steeper decline from 2014 to 2021, where the APC reached −2.90% (Figure 2A).

In the Low SDI region, the trend was less consistent. The overall AAPC was 0.01%, indicating minimal change across the full-time span. A slight decrease was noted between 1990 and 2010 (APC −0.06%), followed by a sharp increase in the burden between 2010 and 2014 (APC 3.53%), and a subsequent decline from 2014 to 2021 (APC −1.74%; Figure 2B). The Low-middle SDI region exhibited a complex trend with an overall AAPC of −0.18%. The initial period from 1990 to 1997 saw a moderate increase (APC 0.43%), which was followed by a decrease from 1997 to 2000 (APC −1.70%). An increase occurred between 2000 and 2014 (APC 0.45%), followed by another decline in the final period from 2014 to 2021 (APC −1.40%; Figure 2C).

The Middle SDI region experienced a relatively stable trend with an overall AAPC of −0.7%. Between 1990 and 2000, the burden decreased slightly (APC −0.35%) before experiencing a sharp increase between 2000 and 2004 (APC 1.78%). From 2007 to 2014, the trend was largely stable (APC 0.20%), but a notable decrease occurred between 2014 and 2019, with an APC of −3.98% (Figure 2D).

In the High-middle SDI region, the overall burden of IHD attributed to PM pollution steadily declined, with an AAPC of −2.06%. The period from 1994 to 2021 witnessed a continuous decline, with the most substantial reduction occurring between 2014 and 2019 (APC −5.03%). However, a smaller decline was observed in the final years, with an APC of −1.10% from 2019 to 2021 (Figure 2E). The High SDI region showed the most significant and consistent decline in IHD burden attributable to PM pollution, with an overall AAPC of −4.75%. The rate of decline was greatest between 2004 and 2011 (APC −6.18%), and although the trend remained negative throughout the study period, the rate of decline slowed slightly in recent years, with an APC of −0.44% between 2019 and 2021. This consistent downward trend highlights the effectiveness of interventions and policies aimed at reducing air pollution and its associated health impacts in higher-income regions (Figure 2F). The specific information of AAPC in Supplementary material 4. The specific information of APC in Supplementary material 5.

### 3.3 Net drift in IHD attributable to PM pollution across SDI levels

**Figure 3:**
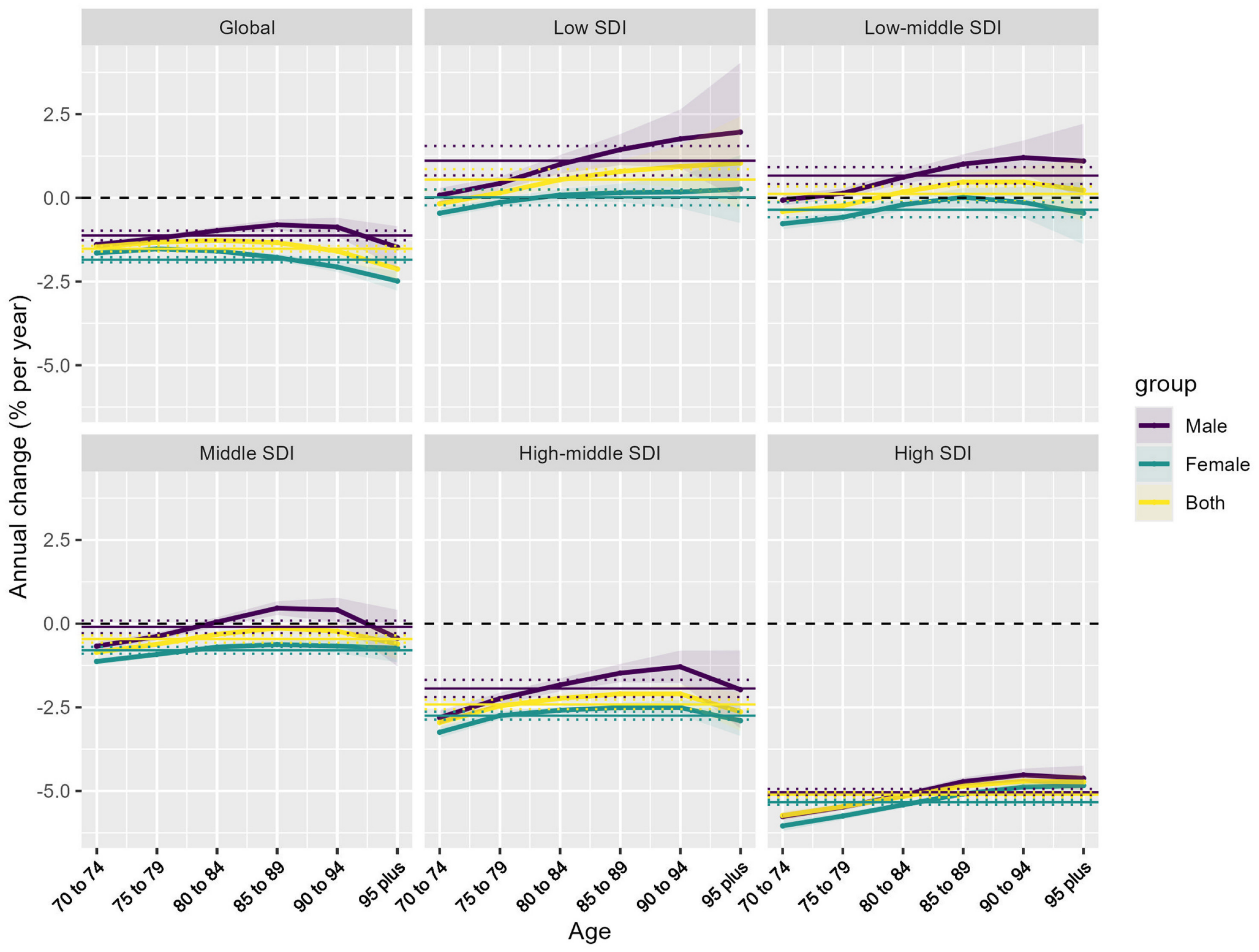
Net drift of IHD attributable to PM pollution by SDI regions and age groups. Net annual percentage change (% per year) in IHD attributable to PM pollution across different SDI regions and age groups, stratified by sex (male, female, and both combined).

In 2021, the global net drift in DALYs associated with IHD attributable to PM pollution varied significantly across SDI levels and between genders. Globally, the net drift for IHD-related DALYs was −1.13% (95% CI, −1.27 to −0.98) in males and −1.85% (95% CI, −1.93 to −1.77) in females, indicating a more significant decline in females. Among SDI regions, the Low SDI region showed the highest growth rate in males, at 1.11% (95% CI, 0.67–1.55), while the High SDI region had the steepest decline, at −5.04% (95% CI, −5.14 to −4.94). For females, the highest growth was observed in the Low SDI region at 0.01% (95% CI, −0.22 to 0.25), and the largest decline was in the High SDI region at −5.34% (95% CI, −5.41 to −5.26). Overall, the combined net drift for both genders was 0.55% (95% CI, 0.24–0.86) in the Low SDI region and −5.11% (95% CI, −5.19 to −5.03) in the High SDI region (Figure 3). The specific information of net drift in Supplementary material 6.

### 3.4 Age, period, and cohort effects on IHD attributable to PM pollution incidence and death rate, 1990–2021

**Figure 4:**
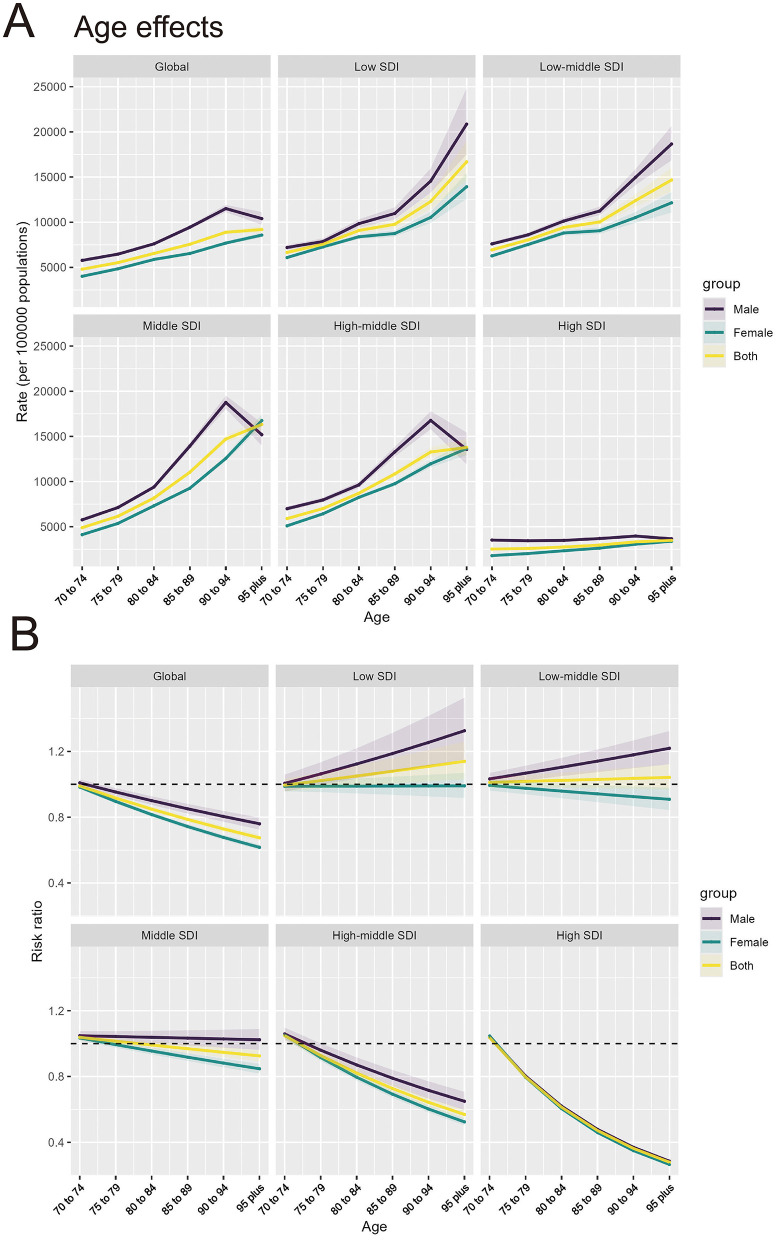
Age effects of IHD attributable to PM pollution by SDI regions and age groups. **(A)** Age effects of IHD attributable to PM pollution (rate per 100,000 population) across SDI regions. **(B)** Risk ratio of IHD attributable to PM pollution across SDI regions by age groups.

**Figure 5:**
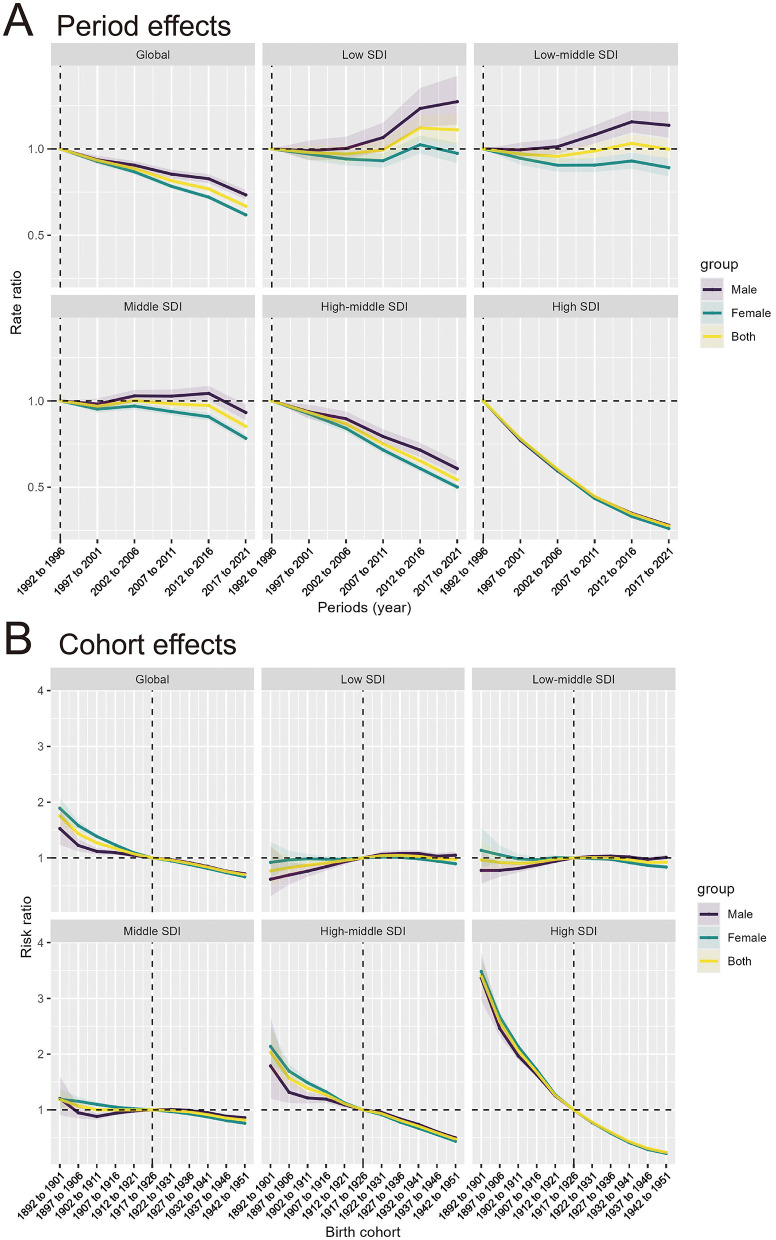
Period and cohort effects of IHD attributable to PM pollution by SDI regions. **(A)** Period effects of IHD attributable to PM pollution across SDI regions by periods. **(B)** Cohort effects of IHD attributable to PM pollution across SDI regions by birth cohorts.

(1) **Age Effect:** The age-specific rates of IHD attributable to PM pollution show a clear increase with advancing age across all SDI regions. Globally, and in the Low and Low-middle SDI regions, IHD rates rise significantly from ages 70 to 95 and older, with males experiencing higher rates than females in all age groups. In these SDI regions, the oldest age groups (90–94 years and 95+ years) display a substantial increase in IHD burden. In contrast, the High SDI regions show a more gradual increase, with a slight peak in the 95+ age group (Figure 4A). Globally, the risk ratio for IHD attributable to PM pollution declines with age. The High and High-middle SDI regions show the sharpest decline in risk ratios with advancing age, indicating a lower relative risk of IHD in older populations in these higher-income regions. However, the risk ratio in Low SDI regions steadily increases with age, especially in males (Figure 4B).
(2) **Period Effect:** The period effect, depicted in Figure 5A, reflects the influence of environmental, healthcare, and social factors on IHD incidence and death rate during specific time periods. Globally, the period effects demonstrate a consistent decline in the rate ratios for IHD attributable to PM pollution from 1990 to 2021. Both males and females exhibit a reduction in rate ratios, with a steeper decline observed after 2016. The High and High-middle SDI regions show the most pronounced decrease, reflecting the impact of improved air quality measures and healthcare advancements over time. The Low SDI region, however, shows an initial period of stability, followed by a slight increase in more recent years, especially among males, suggesting a growing burden in this region.
(3) **Cohort Effect:** The cohort effect, as shown in Figure 5B, captures the influence of birth cohort on the risk of IHD incidence and death rate attributable to PM pollution, representing the evolution of risk in individuals born during specific time periods. Cohort effects show a marked decline in risk ratios for IHD attributable to PM pollution, particularly for cohorts born after 1922–1931 in most SDI regions. Globally and in the High and High-middle SDI regions, there is a consistent downward trend, with more recent birth cohorts (e.g., 1942–1951) exhibiting significantly lower risk ratios compared to earlier cohorts. In the Low and Low-middle SDI regions, the risk ratios show less consistent patterns, with some cohorts (e.g., those born between 1927 and 1936) showing stabilization or slight increases, particularly among males, indicating that risk may persist in these populations.

### 3.5 Driving factors of IHD attributable to PM pollution, 1990–2021

**Figure 6:**
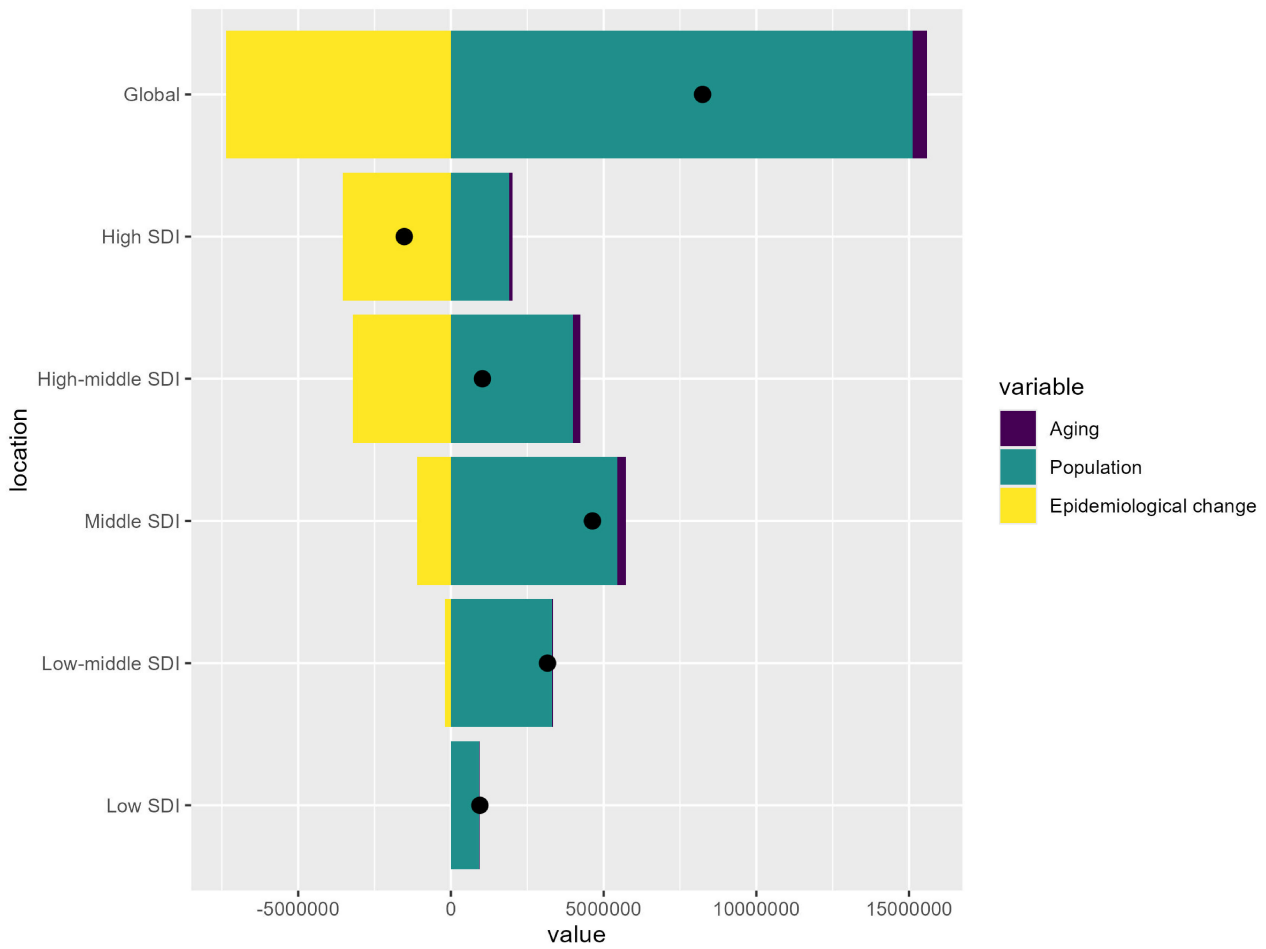
Drivers of IHD attributable to PM pollution by SDI regions. The bar chart shows the relative contributions of aging, population growth, and epidemiological change to the overall IHD burden globally and across different SDI levels.

From 1990 to 2021, there has been a global increase of 8.24 million DALYs attributable to IHD due to PM pollution. Population growth is the largest contributor (183.57%), while aging is also contributing to DALYs (5.73%). In contrast, epidemiological changes have mitigated the burden (−89.29%). In the High SDI region, the IHD burden has decreased by 1.53 million DALYs overall. This decline is primarily driven by epidemiological changes (−232%). In the Low SDI region, the IHD burden increased by 946,343 DALYs. Population growth was the dominant factor (98.64%), while aging added 14,140 DALYs (1.49%). Epidemiological changes had a negligible effect, reducing only 1,297 DALYs (−0.14%; Figure 6). The specific information of driving factors in Supplementary material 7.

### 3.6 Frontiers analysis of IHD DALY rates relative to SDI, 1990–2021

**Figure 7:**
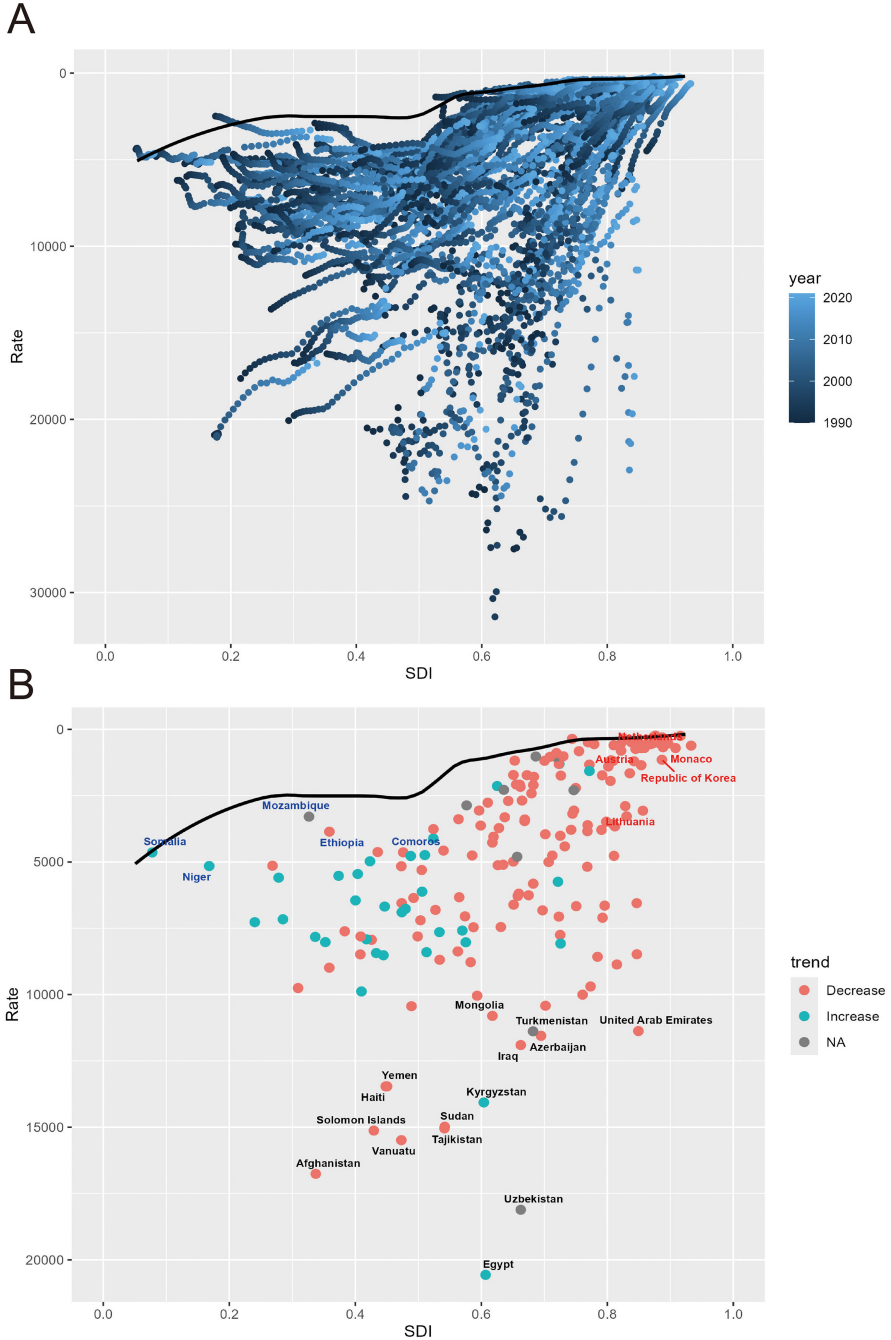
Frontier analysis based on SDI and IHD DALYs rate. **(A)** Frontier analysis based on SDI and IHD DALYs rate from 1990 to 2021. **(B)** Frontier analysis based on SDI and IHD DALYs rate in 2021.

Figure 7A illustrates the changes in IHD DALY rates (vertical axis) over time (depicted by a color gradient from dark blue in 1990 to light blue in 2020) at different SDI levels (horizontal axis). As countries experience economic development, DALY rates generally decline over time. The solid black line represents the optimal (frontier) DALY rate achievable at a given SDI level. Countries that deviate further from this line exhibit greater unrealized health gains, indicating that their performance is suboptimal relative to their level of development. In Figure 7B, the frontier is delineated by a solid black line, and countries and territories are represented as dots. The 15 countries with the largest actual differences from the frontier (largest IHD DALYs gap) are labeled in black, including Yemen, Azerbaijan, Iraq, and Sudan. Five countries with Low SDI but closest to the frontier, such as Somalia, Niger, Mozambique, Ethiopia, and the Comoros, are marked in blue, indicating that despite their low level of development, they are achieving the best possible outcomes within their capacity. In contrast, five countries with High SDI but the largest actual distance from the frontier, including Austria, Netherlands, Republic of Korea, Monaco, and Lithuania, are marked in red, signifying that while they have achieved a high level of development, they have not yet realized their potential in reducing DALY rates and should reconsider how to leverage their advanced resources to further lower these rates. Middle and High-middle SDI regions have greater burden improvement potential. The specific information of frontiers analysis in Supplementary material 8.

### 3.7 Predicted rise in IHD cases and incidence rates from 1990 to 2044

**Figure 8:**
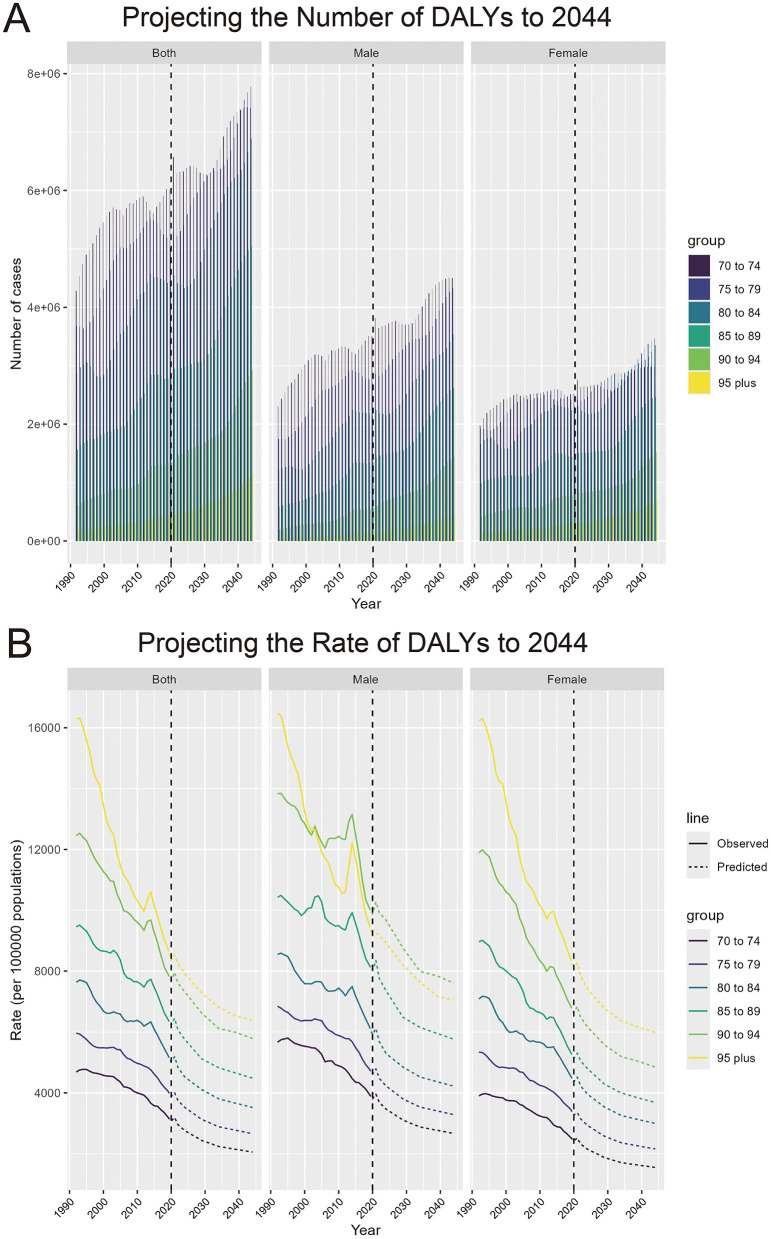
Projecting the number and rate of DALYs due to IHD attributable to PM pollution to 2044. **(A)** Projecting the number of DALYs to 2044, by sex and age groups. **(B)** Projecting the rate of DALYs to 2044 (per 100,000 population), by sex and age groups.

The projections for the DALYs of IHD attributable to PM pollution from 1990 to 2044 show a dramatic rise in the number of DALYs across all age groups over 70 years. After 2020, the number of DALYs is projected to increase significantly. The number of DALYs in the older adult population, particularly those aged over 95, is also expected to rise substantially, although the absolute numbers are smaller compared to younger older adult groups. Both males and females are projected to follow similar patterns of growth in DALY numbers (Figure 8A). In terms of DALY rates, projections show a steady decline across all age groups after 2020, with the most significant decline expected in the oldest population segment (those over 95 years old; Figure 8B).

## 4 Discussion

The results of this study highlight several critical insights regarding the global burden of IHD attributable to PM pollution, revealing notable trends and regional disparities that provide a deeper understanding of how socio-economic factors, demographic shifts, and environmental policies interplay in shaping health outcomes. While the overall global decline in IHD-related DALY rates attributable to PM pollution signals progress in managing both air quality and cardiovascular health, the persistence of large disparities across regions underscores the uneven distribution of these improvements and the significant challenges that remain.

### 4.1 Global decline and regional disparities

The global decline in age-standardized DALY rates for IHD attributable to PM pollution from 1990 to 2021 reflects, in part, the success of international and national efforts to reduce air pollution through stricter environmental regulations and public health interventions (26). This progress is most evident in High and High-middle SDI regions, where significant investments in air quality control, healthcare access, and health education have yielded substantial reductions in the burden of IHD (27). Countries in these regions have benefited from improved healthcare infrastructure, better access to advanced medical treatments for cardiovascular disease, and robust public policies aimed at reducing emissions from industrial activities, transportation, and energy production (28–30). The accelerated decline in High SDI regions, particularly from 2004 to 2011, highlights how coordinated efforts in policy and healthcare can produce marked improvements in public health.

However, the contrasting trends in Low and Low-middle SDI regions reveal a different narrative. In these areas, the overall burden of IHD attributable to PM pollution has been far less responsive to global progress. The slower or inconsistent improvements reflect deep-rooted challenges in these regions, including weaker enforcement of environmental regulations (31), less access to healthcare services (32), and higher exposure to PM pollution, particularly in rapidly urbanizing areas (20). The slight periods of increases in IHD burden during the early 2000s in some of these regions illustrate the detrimental effects of unchecked urbanization and industrialization (33), where economic growth has often occurred without corresponding improvements in air quality management.

### 4.2 Driving forces: population growth, aging, and epidemiological changes

One of the study's most striking findings is the critical role that population growth play in driving the overall burden of IHD attributable to PM pollution. The decomposition analysis reveals that population growth is the largest contributor to the increase in DALYs globally, especially in Low and Low-middle SDI regions, where population growth remains high. As these populations continue to expand, more individuals are exposed to the health risks associated with PM pollution, particularly in densely populated urban areas where pollution levels are typically higher (34, 35).

Aging is also a key factor driving the IHD burden, particularly in Middle SDI regions. As life expectancy increases, the aging population, especially those over 70, experiences heightened susceptibility to cardiovascular diseases (36). The results show that while overall DALY rates have declined, older age groups continue to bear a significant portion of the burden.

In contrast, epidemiological changes, such as better cardiovascular healthcare and reductions in smoking and other risk factors, have played a crucial mitigating role (37, 38), particularly in High SDI regions. These improvements have significantly offset the negative impacts of population growth and aging, contributing to the overall decline in DALY rates in these regions. In Low SDI regions, the lack of significant epidemiological changes means that the rising IHD burden cannot be adequately mitigated, leaving these populations at heightened risk.

### 4.3 Unrealized potential: frontier analysis and policy gaps

The frontier analysis offers a revealing look at the unrealized potential for reducing IHD-related DALYs attributable to PM pollution, particularly in Middle and High-middle SDI regions. Many countries in these regions, despite their relative economic advancement, have not yet fully leveraged their development potential to reduce IHD burden. For example, the Republic of Korea has experienced rapid development and urbanization due to significant economic growth, which has resulted in severe air pollution (39). However, a study found that the emergency reduction measures (ERMs) implemented in response to high concentrations of particulate matter in Seoul were ineffective, and the existing ERM policies in the Republic of Korea are insufficient to effectively reduce PM_2.5_ levels (31).

The identification of countries operating near their frontier in Low SDI regions, such as Somalia and Mozambique, suggests that these countries are already maximizing their health outcomes given their current socio-economic conditions. This finding emphasizes the need for external support and investment in these regions to bridge the resource gap and provide the necessary infrastructure for further health improvements.

### 4.4 Future projections and policy implications

The projections through 2044 offer a cautionary outlook. Although DALY rates are expected to continue declining globally, the absolute number of DALYs is projected to rise, driven by population growth. This trend highlights a potential strain on healthcare systems, especially in middle and low-income countries that may lack the capacity to manage the growing older adult population (40). Therefore, it is necessary to implement targeted interventions for the older adult. These interventions, such as regular cardiovascular screenings (41), increased indoor physical activities, and stricter air quality control policies (42), can enhance the older adult's access to preventive healthcare. Policies aimed at reducing PM pollution, such as transitioning to cleaner energy sources (43), promoting sustainable urbanization (44), and enforcing stricter air quality standards, are essential.

### 4.5 Limitations and future directions

While this study offers a comprehensive analysis of the global burden of IHD attributable to PM pollution, it has several limitations. First, the reliance on large-scale datasets such as the GBD may introduce uncertainties, particularly in regions with limited data availability or reporting inconsistencies. This can affect the accuracy of the DALY estimates and the granularity of regional analyses. Second, the study does not account for the potential impacts of future technological advancements, policy changes, or shifts in energy sources that could alter PM pollution levels and health outcomes. Additionally, the models used for forecasting may not fully capture the complex interactions between demographic shifts, healthcare access, and environmental factors, especially in Low and Low-middle SDI regions.

Future research should focus on improving the accuracy of pollution exposure assessments, particularly in underrepresented regions, and integrating real-time air quality data with health outcomes. There is also a need to explore the long-term effects of emerging pollution sources, such as wildfires and extreme weather events, which could exacerbate IHD risks.

## 5 Conclusion

This study offers a comprehensive evaluation of the global, regional, and national burden of IHD attributable to PM pollution, emphasizing the crucial role of environmental factors in cardiovascular health outcomes. Our findings highlight significant progress in reducing the IHD burden in high SDI regions, where air pollution control measures and healthcare improvements have led to notable reductions in IHD-related DALYs. However, substantial regional disparities remain, with low and low-middle SDI regions experiencing slower progress. These regions face heightened challenges due to factors such as rapid population growth, aging populations, and insufficient healthcare infrastructure, which exacerbate the burden of IHD. One of the most striking findings of this study is the substantial unrealized potential for further reductions in IHD burden in middle SDI regions. These areas, despite having some economic development, still show considerable room for improvement in both environmental and healthcare policies. Strengthening healthcare systems and implementing stricter air quality regulations are crucial steps toward reducing the future burden of IHD in these regions. Furthermore, the study reveals the potential for more equitable health outcomes globally. By addressing the unique challenges faced by low and low-middle SDI regions—particularly in terms of air pollution control and healthcare accessibility—this research supports the development of policies that aim to reduce the health disparities associated with PM pollution. To achieve meaningful progress, it is critical to prioritize the most vulnerable populations, including those in the older adult age group, and to promote policies that target long-term reductions in air pollution exposure.

## Data Availability

The raw data supporting the conclusions of this article will be made available by the authors, without undue reservation.

## References

[1] Pastena P Frye JT Ho C Goldschmidt ME Kalogeropoulos AP. Ischemic cardiomyopathy: epidemiology, pathophysiology, outcomes, and therapeutic options. Heart Fail Rev. (2024) 29:287–99. 10.1007/s10741-023-10377-4 38103139

[2] Nowbar AN Gitto M Howard JP Francis DP Al-Lamee R. Mortality from ischemic heart disease. Circ Cardiovasc Qual Outcomes. (2019) 12:e005375. 10.1161/CIRCOUTCOMES.118.005375 31163980 PMC6613716

[3] de Bont J Jaganathan S Dahlquist M Persson Å Stafoggia M Ljungman P. Ambient air pollution and cardiovascular diseases: an umbrella review of systematic reviews and meta-analyses. J Intern Med. (2022) 291:779–800. 10.1111/joim.13467 35138681 PMC9310863

[4] Xu R Huang S Shi C Wang R Liu T Li Y. Extreme temperature events, fine particulate matter, and myocardial infarction mortality. Circulation. (2023) 148:312–23. 10.1161/CIRCULATIONAHA.122.063504 37486993

[5] Montone RA Rinaldi R Bonanni A Severino A Pedicino D Crea F. Impact of air pollution on ischemic heart disease: evidence, mechanisms, clinical perspectives. Atherosclerosis. (2023) 366:22–31. 10.1016/j.atherosclerosis.2023.01.013 36696748

[6] Chen Q Chen Q Wang Q Xu R Liu T Liu Y. Particulate matter and ozone might trigger deaths from chronic ischemic heart disease. Ecotoxicol Environ Saf. (2022) 242:113931. 10.1016/j.ecoenv.2022.113931 35914398

[7] Oh J Choi S Han C Lee DW Ha E Kim S. Association of long-term exposure to PM(2.5) and survival following ischemic heart disease. Environ Res. (2023) 216:114440. 10.1016/j.envres.2022.114440 36208782

[8] Chanda F Lin KX Chaurembo AI Huang JY Zhang HJ Deng WH. PM(25)-mediated cardiovascular disease in aging: cardiometabolic risks, molecular mechanisms and potential interventions. Sci Total Environ. (2024) 954:176255. 10.1016/j.scitotenv.2024.176255 39276993

[9] Xie Y Tao S Pan B Yang W Shao W Fang X. Cholinergic anti-inflammatory pathway mediates diesel exhaust PM(25)-induced pulmonary and systemic inflammation. J Hazard Mater. (2023) 458:131951. 10.1016/j.jhazmat.2023.131951 37392642

[10] Moufarrej L Verdin A Cazier F Ledoux F Courcot D. Oxidative stress response in pulmonary cells exposed to different fractions of PM(2.5-0.3) from urban, traffic and industrial sites. Environ Res. (2023) 216:114572. 10.1016/j.envres.2022.114572 36244444

[11] Liang S Zhang J Ning R Du Z Liu J Batibawa JW. The critical role of endothelial function in fine particulate matter-induced atherosclerosis. Part Fibre Toxicol. (2020) 17:61. 10.1186/s12989-020-00391-x 33276797 PMC7716453

[12] Tian M Zhao J Mi X Wang K Kong D Mao H. Progress in research on effect of PM(25) on occurrence and development of atherosclerosis. J Appl Toxicol. (2021) 41:668–82. 10.1002/jat.4110 33263192

[13] Chaulin AM Sergeev AK. The role of fine particles (PM 25) in the genesis of atherosclerosis and myocardial damage: emphasis on clinical and epidemiological data, and pathophysiological mechanisms. Cardiol Res. (2022) 13:268–82. 10.14740/cr1366 36405225 PMC9635774

[14] Rajagopalan S Al-Kindi SG Brook RD. Air pollution and cardiovascular disease: JACC state-of-the-art review. J Am Coll Cardiol. (2018) 72:2054–70. 10.1016/j.jacc.2018.07.099 30336830

[15] Karimi A Shirmardi M Hadei M Birgani YT Neisi A Takdastan A. Concentrations and health effects of short- and long-term exposure to PM(2.5), NO(2), and O(3) in ambient air of Ahvaz city, Iran (2014-2017). Ecotoxicol Environ Saf. (2019) 180:542–8. 10.1016/j.ecoenv.2019.05.026 31128552

[16] Faridi S Shamsipour M Krzyzanowski M Künzli N Amini H Azimi F. Long-term trends and health impact of PM(2.5) and O(3) in Tehran, Iran, 2006-2015. Environ Int. (2018) 114:37–49. 10.1016/j.envint.2018.02.026 29477017

[17] Sangkham S Phairuang W Sherchan SP Pansakun N Munkong N Sarndhong K. An update on adverse health effects from exposure to PM25. Environ Adv. (2024) 18:100603. 10.1016/j.envadv.2024.100603

[18] Feng R Wang K Wang F. Quantifying influences of administrative division adjustment on PM(2.5) pollution in China's mega-urban agglomerations. J Environ Manag. (2022) 302:113993. 10.1016/j.jenvman.2021.113993 34715614

[19] Zhang W Cui R Li C Ge H Zhang Z Tang X. Impact of urban agglomeration construction on urban air quality-empirical test based on PSM-DID model. Sci Rep. (2023) 13:15099. 10.1038/s41598-023-42314-8 37700084 PMC10497513

[20] Lim S Bassey E Bos B Makacha L Varaden D Arku RE. Comparing human exposure to fine particulate matter in low and high-income countries: a systematic review of studies measuring personal PM(25) exposure. Sci Total Environ. (2022) 833:155207. 10.1016/j.scitotenv.2022.155207 35421472 PMC7615091

[21] Liu Q Wang S Zhang W Li J Dong G. The effect of natural and anthropogenic factors on PM(25): empirical evidence from Chinese cities with different income levels. Sci. Total Environ. (2019) 653:157–67. 10.1016/j.scitotenv.2018.10.367 30408664

[22] GBD 2021 Diabetes Collaborators. Global, regional, and national burden of diabetes from 1990 to 2021 with projections of prevalence to 2050: a systematic analysis for the Global Burden of Disease Study 2021. Lancet. (2023) 402:203–34. 10.1016/S0140-6736(23)01301-6 37356446 PMC10364581

[23] Zhang H Zhou XD Shapiro MD Lip GYH Tilg H Valenti L. Global burden of metabolic diseases, 1990-2021. Metabolism. (2024) 160:155999. 10.1016/j.metabol.2024.155999 39151887

[24] Chew NWS Ng CH Tan DJH Kong G Lin C Chin YH. The global burden of metabolic disease: data from 2000 to 2019. Cell Metabol. (2023) 35:414–28.e3. 10.1016/j.cmet.2023.02.003 36889281

[25] Foreman KJ Marquez N Dolgert A Fukutaki K Fullman N McGaughey M. Forecasting life expectancy, years of life lost, and all-cause and cause-specific mortality for 250 causes of death: reference and alternative scenarios for 2016-40 for 195 countries and territories. Lancet. (2018) 392:2052–90. 10.1016/S0140-6736(18)31694-5 30340847 PMC6227505

[26] Li C van Donkelaar A Hammer MS McDuffie EE Burnett RT Spadaro JV. Reversal of trends in global fine particulate matter air pollution. Nat Commun. (2023) 14:5349. 10.1038/s41467-023-41086-z 37660164 PMC10475088

[27] Broome RA Fann N Cristina TJ Fulcher C Duc H Morgan GG. The health benefits of reducing air pollution in Sydney, Australia. Environ Res. (2015) 143:19–25. 10.1016/j.envres.2015.09.007 26414085

[28] Chien F Sadiq M Nawaz MA Hussain MS Tran TD Le Thanh T. step toward reducing air pollution in top Asian economies: The role of green energy, eco-innovation, and environmental taxes. J Environ Manage. (2021) 297:113420. 10.1016/j.jenvman.2021.113420 34333309

[29] Timmis A Vardas P Townsend N Torbica A Katus H De Smedt D. European Society of Cardiology: cardiovascular disease statistics 2021. Eur Heart J. (2022) 43:716–99. 10.1093/ehjqcco/qcac014 35016208

[30] Chien F Ananzeh M Mirza F Bakar A Vu HM Ngo TQ. The effects of green growth, environmental-related tax, and eco-innovation towards carbon neutrality target in the US economy. J Environ Manage. (2021) 299:113633. 10.1016/j.jenvman.2021.113633 34492439

[31] Ho CH Kim KY. Ineffective implementation of emergency reduction measures against high concentrations of particulate matter in Seoul, Republic of Korea. Environ Monit Assess. (2023) 195:1127. 10.1007/s10661-023-11754-0 37650945 PMC10471636

[32] Kalisa E Clark ML Ntakirutimana T Amani M Volckens J. Exposure to indoor and outdoor air pollution in schools in Africa: current status, knowledge gaps, and a call to action. Heliyon. (2023) 9:e18450. 10.1016/j.heliyon.2023.e18450 37560671 PMC10407038

[33] Sun J Zhou T Wang D. Effects of urbanisation on PM(25) concentrations: a systematic review and meta-analysis. Sci Total Environ. (2023) 900:166493. 10.1016/j.scitotenv.2023.166493 37619722

[34] Chowdhury S Dey S Smith KR. Ambient PM(2.5) exposure and expected premature mortality to 2100 in India under climate change scenarios. Nat Commun. (2018) 9:318. 10.1038/s41467-017-02755-y 29358713 PMC5778135

[35] Fu Z Li R. The contributions of socioeconomic indicators to global PM(25) based on the hybrid method of spatial econometric model and geographical and temporal weighted regression. Sci Total Environ. (2020) 703:135481. 10.1016/j.scitotenv.2019.135481 31759707

[36] Moturi S Ghosh-Choudhary SK Finkel T. Cardiovascular disease and the biology of aging. J Mol Cell Cardiol. (2022) 167:109–17. 10.1016/j.yjmcc.2022.04.005 35421400

[37] Jilani MH Javed Z Yahya T Valero-Elizondo J Khan SU Kash B. Social determinants of health and cardiovascular disease: current state and future directions towards healthcare equity. Curr Atheroscler Rep. (2021) 23:55. 10.1007/s11883-021-00949-w 34308497

[38] Duncan MS Freiberg MS Greevy RA Jr., Kundu S, Vasan RS, Tindle HA. Association of smoking cessation with subsequent risk of cardiovascular disease. JAMA. (2019) 322:642–50. 10.1001/jama.2019.10298 31429895 PMC6704757

[39] Park J Kim H Kim Y Heo J Kim SW Jeon K. Source apportionment of PM(25) in Seoul, South Korea and Beijing, China using dispersion normalized PMF. Sci Total Environ. (2022) 833:155056. 10.1016/j.scitotenv.2022.155056 35395292

[40] McMaughan DJ Oloruntoba O Smith ML. Socioeconomic status and access to healthcare: interrelated drivers for healthy aging. Front Public Health. (2020) 8:231. 10.3389/fpubh.2020.00231 32626678 PMC7314918

[41] Ermolao A Gasperetti A Rigon A Patti A Battista F Frigo AC. Comparison of cardiovascular screening guidelines for middle-aged/older adults. Scand J Med Sci Sports. (2019) 29:1375–82. 10.1111/sms.13457 31059145

[42] He G Jiang M Tian S He L Bai X Chen S. Clean air policy reduces the atherogenic lipid profile levels: Results from China Health Evaluation And risk Reduction through nationwide Teamwork (ChinaHEART) Study. J Hazard Mater. (2024) 478:135394. 10.1016/j.jhazmat.2024.135394 39128148

[43] Wang J Du W Lei Y Duan W Mao K Wang Z. Impacts of household PM(25) pollution on blood pressure of rural residents: implication for clean energy transition. Sci Total Environ. (2023) 884:163749. 10.1016/j.scitotenv.2023.163749 37120026

[44] Bouscasse H Gabet S Kerneis G Provent A Rieux C Ben Salem N. Designing local air pollution policies focusing on mobility and heating to avoid a targeted number of pollution-related deaths: Forward and backward approaches combining air pollution modeling, health impact assessment and cost-benefit analysis. Environ Int. (2022) 159:107030. 10.1016/j.envint.2021.107030 34890901

