# Supplementary material for: Global, regional, and national burden of IHD attributable to PM pollution aged 70 and above: an age-period-cohort modeling and frontiers analysis study
Source: Front Public Health. 2025 Jun 4;13:1573599. doi: 10.3389/fpubh.2025.1573599 (PMC12174465; doi:10.3389/fpubh.2025.1573599)
Supplement: Supplementary file 8 [file Table_1.docx]

**Supplementary Methods**

**1.Joinpoint regression analysis**

Time trend analysis is a vital aspect of epidemiological research. However, traditional regression models often fall short in capturing localized variations, as they tend to emphasize overall trends across the entire study period. To address this limitation, Kim et al. introduced the Joinpoint regression model in 1998. This model utilizes a segmented regression approach, breaking the time period into multiple segments by identifying specific Joinpoints. Each segment is then analyzed separately, offering a more nuanced understanding of disease trends during particular intervals within the study period^1^. Developed by the Division of Cancer Control and Population Sciences at the U.S. National Cancer Institute, the Joinpoint regression model has since become a key method for analyzing patterns in disease incidence and mortality rates, providing insights that are often missed by traditional models.

**(I) Model Introduction**

The Joinpoint regression model presents two variations: the linear model (y = xb) and the logarithmic linear model (ln y = xb). The choice between these models depends on the distribution of the dependent variable and the nature of the data. The linear model is generally preferred when the dependent variable has a normal or approximately normal distribution, and the sample size is sufficiently large, typically over 100 observations. It is particularly well-suited for analyzing continuous variables, such as height or weight. On the other hand, the log-linear model is more appropriate when the dependent variable follows an exponential or Poisson distribution. This scenario is often encountered in epidemiological research involving population-based data, such as incidence rates or case counts. For instance, when analyzing trends in metrics related to thalassemia (such as incidence, prevalence, mortality rates, or DALYs) the log-linear model is typically applied, as it better accounts for the nature of the data distribution in these cases.

**(II) Modeling Method**

The Joinpoint regression model primarily utilizes the grid search method (GSM) as its default modeling approach. In this method, the study data is divided into a grid, with each intersection representing a possible scenario for the analysis^1^. For each scenario within specified intervals, GSM calculates performance metrics for the corresponding equations using a fixed step size to identify the optimal function. Essentially, the model employs GSM to evaluate all potential Joinpoints (points where segments of the data meet) and computes metrics such as the sum of squares errors (SSE) and mean squared errors (MSE) for each candidate. The grid point with the smallest MSE is selected as the Joinpoint. Based on these identified Joinpoints and the respective interval functions, the model then fits the parameters of the equation, including β_0_, β_1_, δ_1_, ..., δ_k_^2^.

**(III) Model Optimization**

In the Joinpoint software, model optimization is primarily achieved using the Monte Carlo permutation test. Prior to initiating the modeling process, the range for the number of Joinpoints, k ∈ (MIN, MAX), where MIN is typically set to 0, indicating the minimum number of Joinpoints, and MAX represents he upper limit on the allowable number of Joinpoints. The test evaluates the null hypothesis (H_0_: the number of Joinpoints is k = k_a_) against the alternative hypothesis (H_1_: the number of Joinpoints is k = k_b_). The procedure begins with k_a_=MIN and k_b_=MAX. If H_0_ is rejected, the value of k is incremented to k_a_ + 1 for further testing. If H_0_ is not rejected, k is reduced to k_b_ - 1. This iterative process continues until ka equals k_b_, indicating that the value of k = k_a_ = k_b_ is the optimal number of Joinpoints selected by the permutation test, resulting in the best-fitting model^3^.

**(IV) Index Calculation**

In the Joinpoint model, two primary outcome indicators are used to describe trends over time: the Annual Percent Change (APC) and the Average Annual Percent Change (AAPC). These indicators are typically reported with their 95% confidence intervals (CI) to provide a measure of uncertainty. The APC quantifies the year-to-year percentage change in a dependent variable, such as an incidence rate, over a specified time period. It is particularly useful for identifying trends and how they evolve across different segments of time. For instance, in a logarithmic linear model expressed as ln (y) =β_0_ + β_1_ x, where y is the incidence rate and x represents the year, the APC can be calculated from the fitted model using the formula:


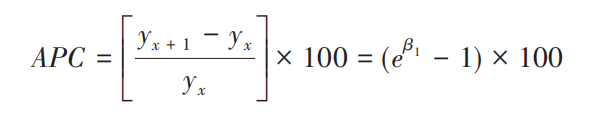


The lower and upper limits of the 100(1-α) % confidence interval are respectively:


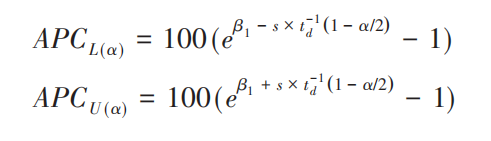


The regression coefficient is represented by β_1_ in the above formula, the standard error of β_1_ is indicated by s, the degrees of freedom are denoted by d, and the critical value td(q) is the value that corresponds to the qth percentile (e.g., 95%) of the t-distribution with d degrees of freedom.

When evaluating a segmented function, the APC is utilized to evaluate the trend inside each independent interval or, in the absence of joinpoints, to study the overall trend. On the other hand, the AAPC is utilized when assessing the overall average trend over several segments. The regression coefficients from each segment are weighted and averaged, with the weights determined by the width (w) of each segment interval, in order to get the AAPC. The following is its formula:


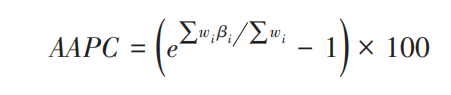


The lower and upper limits of the 100(1-α) % confidence interval are respectively:


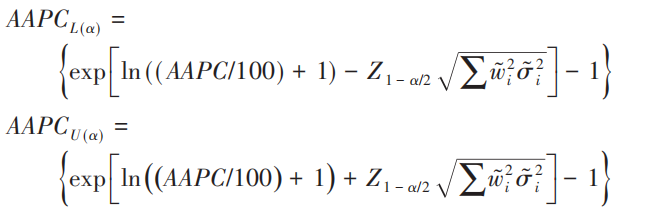


The variable w_i_, which represents the total number of years included in each segment function interval, is specified in the above equation as its span, or duration. The regression coefficient for each interval is denoted by the symbol β_i_, and its variance is represented by σ^2^i. Moreover, Z_α_ corresponds to the value at the α percentile within the normal distribution.

**(V) Software Download**

We went to https://surveillance.cancer.gov/Joinpoint/download to obtain the Joinpoint program from the National Cancer Institute's website. We were able to successfully download the software after registering and giving the required application details. The following is the software's citation for reference: "Joinpoint Regression Program, Version 4.9.1.0 - April 2022; Statistical Methodology and Applications Branch, Surveillance Research Program, National Cancer Institute."

**2.Age-period-cohort modelling analysis**

T2DM mortality estimates and population statistics from different nations and areas were used as input data for the APC model for the GBD 2021. Using this model, the overall temporal trends in mortality were calculated, which were stated as the net drift, or the annual percentage change in mortality, expressed as a percentage per year. The trend due to calendar time and the trend attributable to subsequent cohorts make up the two main components of the net drift. Additionally, the APC model offered estimates of the temporal trends in mortality within particular age groups. These estimates were expressed as the local drift, which reflected the impact of birth cohort effects, or as the annual percentage change of age-specific mortality^4^. A change in mortality of ±1% per year is deemed significant^4^ correlating to changes of approximately ±10%, ±18%, and ±26% in the mortality rate over 10, 20, and 30 years, respectively. Using a Wald chi-squared test, the significance of these yearly percentage changes was assessed^4^. In addition, the outputs of the APC model included adjusted longitudinal age-specific rates for the reference cohort to demonstrate age-related natural trends (i.e., age effects), as well as period (cohort) relative risks of mortality for each period (cohort) to indicate period (cohort) effects^4^. We calculated relative risk by comparing age-specific rates within each cohort to those in a reference cohort, while both sets of rate ratio curves fully capture net drift. The choice of reference cohort is arbitrary and has no impact on result interpretation. Statistical tests were two-sided with a significance level set at p < 0.05, and analysis was carried out using R software version 3.6.3 for this study^5^.

**3.Decomposition analysis**

We initially applied the decomposition methodology developed by Das Gupta^6-8^ to break down the DALYs associated with T2DM into components attributable to changes in the population age structure, population growth, and epidemiologic shifts. The calculation of DALYs for each location was based on the specific formula outlined below:

DALY _ay, py, ey_ = $\sum_{i=1}^{20} ($a _i, y_ * p _y_ * e _i, y_)

Among them, DALY _ay, py, ey_ denotes DALY based on age structure, population, and DALY rate factors for a particular year y; a _i_ _y_ represents the proportion of the population in age group i out of the 20 age groups in given year y; p _y_ denotes the total population in given year y; and e _i, y_ denotes DALYs rate given age category i in year y.

**4.Frontier analysis**

To assess the relationship between the burden of ischemic heart disease (IHD) and socio-demographic development, we employed frontier analysis as a quantitative approach. This method identifies the lowest achievable age-standardized DALY rates based on the development level, as measured by the SDI. The DALYs frontier represents the minimum DALYs that could be reached by each country or territory according to its SDI level. The difference between a country's DALYs and this frontier is called the effective difference. A large effective difference suggests that there are potential improvements (i.e., reductions in IHD DALYs) that could be achieved given the country's position on the development spectrum. To create the frontier for age-standardized IHD DALYs in relation to SDI^9^, we applied data envelopment analysis using the free disposal hull method, allowing for the creation of a non-linear frontier. This analysis utilized data spanning from 1990 to 2020. To account for uncertainty, we generated 1,000 bootstrapped samples by randomly sampling with replacement from the data of all countries and territories across the years. For each SDI value, the mean IHD DALYs was calculated from these bootstrapped samples. A LOESS regression with a local polynomial of degree 1 and a span of 0.2 was applied to produce a smoothed frontier^9^. To mitigate the influence of outliers, countries that were identified as super-efficient were excluded from the frontier generation process^9^. To evaluate the relationship between age-standardized IHD DALY rates and the frontier for the year 2020, we calculated the effective difference, defined as the absolute distance between a country’s 2020 IHD DALY rate and the frontier, based on its SDI in that year. Countries or territories with DALYs lower than the frontier was assigned an effective difference of zero.

**5. Nordpred prediction model**

The Nordpred prediction model, derived from the APC framework, effectively forecasts future trends in DALYs. This model takes into account the interplay between time series data and demographic factors, such as shifts in population structure, disease patterns, and generational effects. To project trends, the Nordpred APC model was employed to estimate DALYs from 2022 to 2044. The Nordpred analysis is conducted in five-year intervals of age, period, and cohort, serving as the foundation for projecting trend data for each period. It utilizes a log-linear age-period-cohort model designed to predict the number or rate of new cases, which helps to moderate exponential growth and constrain linear trend projections to align with recent trends, demonstrating effectiveness in forecasting future burden trends. The model extrapolates from the most recent five years of observed data (three or four years, depending on data availability) using a power function to temper growth. Subsequently, the linear trend from the previous decade is adjusted for the second, third, and fourth prediction periods, either diminishing or enhancing it by 25%, 50%, and 75%, respectively. The final predictions for DALYs in 2044 are calculated by averaging the projected incidence rates for the last two prediction periods, centered on the year 2044^10,11^. The Nordpred software package, implemented in R language (version 4.3.1), integrates dynamic changes in incidence rates and population structure.

**References**

1. Kim HJ, Fay MP, Feuer EJ, Midthune DN. Permutation tests for joinpoint regression with applications to cancer rates. *Stat Med* 2000;**19**:335-351. doi: 10.1002/(sici)1097-0258(20000215)19:3<335::aid-sim336>3.0.co;2-z

2. Siegel RL, Miller KD, Fuchs HE, Jemal AJCCJC. Cancer statistics, 2021. 2021;**71**:7-33. doi:

3. Yang JJ, Trucco EM, Buu A. A hybrid method of the sequential Monte Carlo and the Edgeworth expansion for computation of very small p-values in permutation tests. *Stat Methods Med Res* 2019;**28**:2937-2951. doi: 10.1177/0962280218791918

4. Rosenberg PS, Check DP, Anderson WF. A web tool for age-period-cohort analysis of cancer incidence and mortality rates. *Cancer Epidemiol Biomarkers Prev* 2014;**23**:2296-2302. doi: 10.1158/1055-9965.Epi-14-0300

5. %J RCT. A language and environment for statistical computing. 2021. doi:

6. P. DG. Standardization and decomposition of rates: a user’s manual, Pages 19-36; 1993. p19-36.

7. Das Gupta P. Standardization and decomposition of rates from cross-classified data. *Genus* 1994;**50**:171-196. doi:

8. Chevan A, Sutherland M. Revisiting Das Gupta: refinement and extension of standardization and decomposition. *Demography* 2009;**46**:429-449. doi:

9. Healthcare Access and Quality Index based on mortality from causes amenable to personal health care in 195 countries and territories, 1990-2015: a novel analysis from the Global Burden of Disease Study 2015. *Lancet* 2017;**390**:231-266. doi: 10.1016/s0140-6736(17)30818-8

10. Arnold M, Park JY, Camargo MC*, et al.* Is gastric cancer becoming a rare disease? A global assessment of predicted incidence trends to 2035. *Gut* 2020;**69**:823-829. doi: 10.1136/gutjnl-2019-320234

11. Jürgens V, Ess S, Cerny T, Vounatsou P. A Bayesian generalized age-period-cohort power model for cancer projections. *Stat Med* 2014;**33**:4627-4636. doi: 10.1002/sim.6248
